# Western Immunoblotting for the Diagnosis of *Enterococcus faecalis* and *Streptococcus gallolyticus* Infective Endocarditis

**DOI:** 10.3389/fcimb.2019.00314

**Published:** 2019-09-12

**Authors:** Florent Arregle, Frédérique Gouriet, Bernard Amphoux, Sophie Edouard, Hervé Chaudet, Jean-Paul Casalta, Gilbert Habib, Pierre-Edouard Fournier, Didier Raoult

**Affiliations:** ^1^Aix Marseille Univ., IRD, AP-HM, MEPHI, Marseille, France; ^2^Service de Cardiologie, Hôpital de la Timone, Marseille, France; ^3^Microbiology Laboratory, Institut Hospitalo-Universitaire (IHU) Mediterranée Infection, Marseille, France; ^4^Aix Marseille Univ., IRD, AP-HM, SSA, VITROME, IHU Méditerranée Infection, Marseille, France; ^5^CNR des Rickettsies, fièvre Q, Bartonella, IHU Méditerranée Infection, Marseille, France

**Keywords:** blood culture negative endocarditis, *Enterococcus faecalis*, *Streptococcus gallolyticus*, Western immunoblotting, serology

## Abstract

Blood culture-negative endocarditis (BCNE) remains a diagnostic challenge. In our center, despite a systematic and exhaustive microbiological diagnostics strategy, 22% of patients with BCNE remain without an identified etiology. In an effort to determine the relevance of using Western blot (WB) for the etiological diagnosis of BCNE in patients with early antibiotic use, we developed specific assays for the major infective endocarditis (IE) causative agents, namely, *Staphylococcus aureus, Enterococcus faecalis, Streptococcus anginosus*, and *Streptococcus gallolyticus*. Our technique was effective to identify the antigenic profiles of the four tested agents, but cross-reactions with *S*. *aureus* and *S*. *anginosus* antigens were frequent. A scoring method was developed for the diagnosis of *E. faecalis* and *S. gallolyticus* IE using the presence of reactivity to at least two antigenic bands for each bacterium and the positivity to at least one of the Ef300, Ef72, or Ef36 proteic bands for *E. faecalis*, and positivity for the two Sg75 and Sg97 proteic bands for *S. gallolyticus*. We tested these diagnostic criteria in a prospective cohort of 363 patients with suspected IE. Immunoblotting for the diagnosis of *E. faecalis* IE showed a sensitivity of 100% and a specificity of 99%. The positive and negative predictive values were 73 and 100%, respectively. Regarding *S. gallolyticus* infection, immunoblot had a sensitivity of 100% and a specificity of 95%. However, the positive predictive value was 22%, whereas the predictive negative value was 100%. Using WB, we identified a potential etiological agent in 4 of 14 BCNE cases with no identified pathogen. In conclusion, WB constitutes a promising and helpful method to diagnose *E. faecalis* or *S. gallolyticus* IE in patients with early antibiotic uptake and negative blood cultures.

## Introduction

Blood culture-negative endocarditis (BCNE) remains a diagnostic challenge. Blood cultures remain sterile in 2.5–70% of infectious endocarditis (IE) cases, depending on geographical and epidemiological factors, prior antibiotic use, and non-infective etiologies (Fournier et al., 2010). Our institute is a reference center for the diagnosis of BCNE (Fournier et al., 2017). In order to reduce the proportion of BCNE without etiology, we have developed over the years diagnostic guidelines and progressively implemented new diagnostic methods. The initial step, in 1993, was the systematic testing for rheumatoid factor as well as antibodies to fastidious pathogens including *Coxiella burnetii, Bartonella* spp., *Mycoplasma pneumoniae, Chlamydia pneumoniae*, and *Aspergillus* sp. (Raoult et al., 2005). At the same time, we implemented broad-range 16S rRNA PCR from valvular biopsies and/or blood. In 2003, we added Western immunoblotting (WB) for *Bartonella* spp., including in patients for whom *Bartonella* IFA was negative (Houpikian and Raoult, 2003). In 2010, the kit was enriched with specific real-time PCR assays from cardiac valves and/or blood for *Bartonella* species, *C. burnetii, Enterococcus faecalis, E. faecium, Escherichia coli, Staphylococcus aureus, Streptococcus gallolyticus, S. oralis*, and *Tropheryma whipplei*, increasing the diagnostic efficiency by 24.3% (Fournier et al., 2017). The same year, the diagnostic strategy was completed with determination of antinuclear, anti-DNA, and anti-cardiolipin antibodies as well as immunoglobulin E to pork when other assays failed to provide a diagnosis (Fournier et al., 2011). Each of the above-cited implementation steps resulted in a significant rate of new diagnoses. However, despite these efforts, in our latest study of BCNE, 22% of patients with BCNE, i.e., 4.2% of all IE cases, remained without an identified etiology (Fournier et al., 2017). Recently, as part of our systematic and prospective microbiological testing of patients with IE, we came across two cases of definite IE due to *E. faecalis* documented by positive blood cultures associated with WB profiles in favor of *Bartonella* infection. Initially, we concluded that these were co-infections. However, in the absence of a consistent epidemiological context for *Bartonella* infection, and in the presence of negative immunofluorescence serologies and molecular tests on blood and serum, the possibility of cross-reactions between *E*. *faecalis* and *Bartonella* spp. was documented (see below). Subsequently, we launched an investigation as to whether the diagnosis of *E. faecalis* IE could be obtained using immunoblotting.

In this study, in an effort to determine the relevance of using WB for the etiological diagnosis of BCNE in patients with early antibiotic use, we developed specific WB assays for some important IE causative agents, namely, *S. aureus, E. faecalis, S. anginosus*, and *S. gallolyticus*. A scoring method was developed for the diagnosis of *E. faecalis* and *S. gallolyticus* IE and evaluated in a cohort of patients with suspected IE.

## Materials and Methods

### Index Cases

Case 1: In January 2018, an 83-year-old man presented with a definite *E. faecalis* IE on implanted cardioverter defibrillator without secondary embolism. Three blood cultures grew *E. faecalis*. He also had a *Bartonella*-positive WB. The transesophageal echography showed a 3 cm vegetation on a lead and severe tricuspid insufficiency. He was administered intravenous amoxicillin and ceftriaxone for a period of 6 weeks, and the triple-chamber defibrillator and two abandoned leads were extracted percutaneously. Re-implantation of an epicardial stimulator was performed secondarily. He had a past history of bladder neoplasia in 2014 that had been surgically treated by the Bricker procedure and a nephrostomy with multiple subsequent urinary tract infections. The patient was declared cured after 1-year of follow-up.

Case 2: In February 2018, a 73-year-old man was admitted for *E. faecalis* IE on his native aortic valve complicated with moderate aortic insufficiency, renal embolism, ischemic stroke, and T10–T11 spondylodiscitis. *E. faecalis* was isolated in three blood cultures. He received intravenous amoxicillin and ceftriaxone for a period of 6 weeks. The patient was declared cured after 1-year of follow-up.

**Figure 1:**
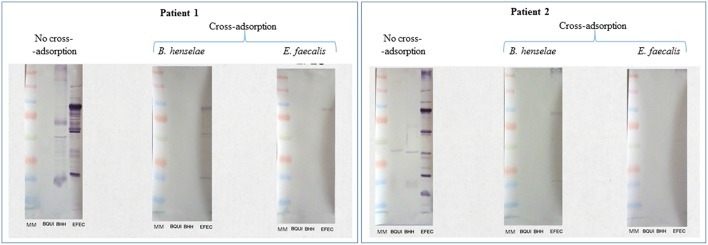
Serological cross-reactions between *E. faecalis* and *Bartonella* spp. in two patients with *E. faecalis* IE. *Bartonella henselae* Houston-1 (ATCC 49882), *B. quintana* Oklahoma (ATCC VR-51-694), and *Enterococcus faecalis* (CSUR P6219) were used as antigens. Both patients showed a strong response to *Bartonella* sp. and *E. faecalis* antigens. Cross-adsorption with *Bartonella henselae* removed the antibody response to *Bartonella* sp. only, confirming the cross-reactivity with *E. faecalis*.

Both patients were diagnosed with *E. faecalis* IE (3/3 positive blood cultures) and also exhibited a positive WB for *Bartonella henselae* and *B. quintana* antigens. The patients' sera also demonstrated a strong IgG/IgM response to *E. faecalis* antigens by WB (Figure 1). Adsorption of the patients' sera with *E. faecalis* antigens removed antibodies to both *Bartonella* and *E. faecalis*, whereas adsorption with *B. henselae* or *B. quintana* antigens removed antibodies to *Bartonella* only, thus confirming cross-reactivity with *Bartonella* sp. antigens and not co-infection.

### Patients and Sera

All patients with clinical suspicion of IE had a standardized diagnostic kit (Fournier et al., 2010) including blood cultures, serological testing for fastidious bacteria (Raoult et al., 2005), immunological blood tests, and, in case of BCNE, WB for *Bartonella* sp. antigens (Houpikian and Raoult, 2003) and PCR from EDTA blood, as described above (Fournier et al., 2017). The diagnosis of IE was based on Duke's modified criteria (Li et al., 2000) and the ESC guidelines (Habib et al., 2015).

**Figure 2:**
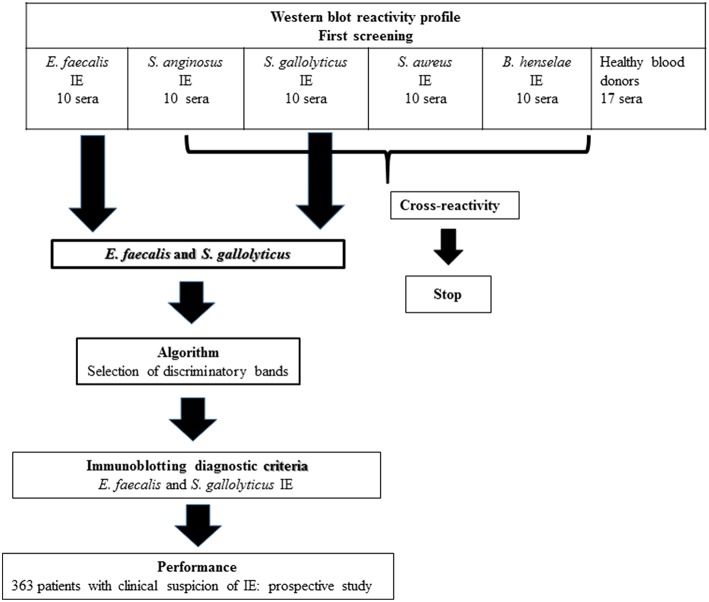
Study design.

Fifty patients with definite IE and an identified etiologic agent were retrospectively selected in our database (Figure 2), including 10 patients each diagnosed with *E. faecalis, S. anginosus, S. gallolyticus, S. aureus*, and *B. henselae* IE. As negative controls, we used sera from 17 healthy blood donors.

At another moment in time, from March to November 2018, we tested prospectively and blindly 363 sera obtained from patients with clinical suspicion of IE hospitalized in La Timone hospital, Marseille.

### Bacterial Strains Used for WB

To obtain a WB antigen, we used the reference strains *B. henselae* Houston-1 (ATCC 49882) (Houpikian and Raoult, 2003). *E. faecalis* strain CSUR P6219, *S. gallolyticus* strain CSUR P6220, *S. anginosus* strain CSUR P6221, and *S. aureus* strain CSUR P6222 that was isolated from blood cultures of patients with IE (CSUR = Collection de souches de l'Unité des Rickettsies, WDCM 875).

### Antigen Preparation

Bacteria were grown on 5% sheep blood-enriched Columbia agar (Biomérieux, Marcy l'Etoile, France) at 37°C in a 5% CO_2_ atmosphere. After 24 h of incubation for *E. faecalis, S. gallolyticus, S. anginosus*, and *S. aureus*, and 7 days of incubation for *B. henselae*, bacteria were harvested and suspended in sterile distilled water prior to being frozen at −20°C.

### WB Analysis

**Figure 3:**
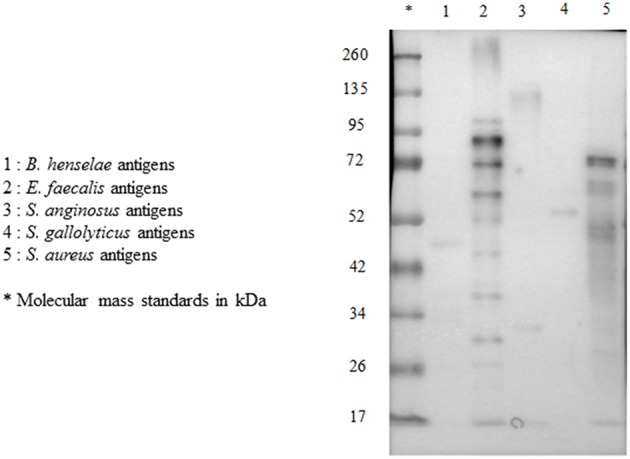
Multiplex immunoblot testing reactivity to *B. henselae, E. faecalis, S. anginosus, S. gallolyticus*, and *S. aureus* in a patient with *E. faecalis* IE.

For each serum, we performed multiplex immunoblot, testing reactivity to antigens from five pathogens: *E. faecalis, S. gallolyticus, S. anginosus, S. aureus*, and *B. henselae* (Figure 3).

For each species, bacterial cells were suspended in sterile distilled water and adjusted to 1 mg of protein/ml spectrophotometrically. A volume of antigen was mixed with a volume of Laemmli solubilizer, as previously reported (Maurin et al., 1997), and the mixture was boiled for 15 min. Eight microliters of the preparation was electrophoresed at 100 V for 2 h through 10% polyacrylamide separating gels with 5% polyacrylamide stacking gels with a Mini Trans-blot cell apparatus (Bio-Rad, Hercules, Calif.). A mixture of pre-stained molecular mass standards (Bio-Rad) was used to estimate the molecular masses of separated antigens. Resolved antigens were then transferred to a 0.45 μm-pore size nitrocellulose membrane (Bio-Rad) for 90 min at 15°C and 100 V. The blots were blocked overnight at 4°C with 5% non-fat milk powder in TBS buffer and washed with distilled water. Sera (diluted 1:200 in TBS−0.5% non-fat milk powder) were applied to the blots for 1 h at room temperature. After three 10 min washes in TBS−0.5% non-fat milk powder, the blots were incubated for 1 h with peroxidase-conjugated goat anti-human IgA, IgG, and IgM (Jackson ImmunoResearch) diluted 1:1000 in TBS−0.5% non-fat milk powder. The blots were washed three times in TBS, and bound conjugate was revealed by incubation with an ECL Western Blotting Substrate (peroxide solution and luminol enhance solution) solution (Promega). Blots were analyzed with a Fusion Fx chemiluminescence imaging system and images were obtained using the Fusion software (Vilber). Protein bands were read with ImageQuant TL (General Electric).

Blots were assessed blindly by the same individual to minimize any variation in the interpretation.

### Statistical Analysis

Statistical analysis was performed using R [R Core Team (2018). R: A language and environment for statistical computing. R Foundation for Statistical Computing, Vie]. For detecting the WB bands allowing for the discrimination of causative species, we used the CHi-square Automatical Interaction Detector (CHAID) (Kass, 1980), which builds decision trees with multiway splits upon categorical dependent and explanatory variables. All decisions based on statistical tests were taken using a maximum alpha risk of 5%.

## Results

### Analysis of WB Reactivity Profile for Each Tested Pathogen

We evaluated the WB reactivity profile for *E. faecalis, S. gallolyticus, S. anginosus*, and *S. aureus* IE using 50 sera from our IE database (Figure 2) and 17 healthy blood donors. The first screening showed that the test was not specific for *S. aureus* and *S. anginosus*, with a high rate of false positive [52/57 (91%) and 47/57 (82%) of the control sera, respectively]. Among the 10 antigenic bands identified for *S. anginosus* and 18 for *S. aureus*, none was specific.

However, *E. faecalis* immunoblotting performed with the 10 sera from patients with *E. faecalis* IE allowed us to identify 14 protein bands (Table 1). All patients showed reactivity to at least two different antigenic bands. In addition, when compared to immunoblot performed using 40 sera from patients with non-enterococcal IE, we identified four antigenic bands (Ef300, Ef39, Ef36, and Ef23) with a 100% specificity and two antigenic bands, namely, Ef 89 and Ef 59, with the best sensitivity (100 and 80%, respectively). Among healthy blood donors, only one patient showed reactivity to two antigenic bands (Ef26 and Ef16).

**Table 1:** Western blot (WB) pattern of reactivity of sera from *E. faecalis* infective endocarditis.

Protein band^*^	**Number of sera with WB reactivity to protein band**		**Sensitivity (%)**	**Specificity (%)**
	***E. faecalis* IE (*n* = 10)**	**Controls (*n* = 57)**		
Ef300	5	0	50	100
Ef99	7	5	70	91
Ef89	10	5	100	91
Ef72	5	1	50	98
Ef59	8	6	80	89
Ef52	4	4	40	93
Ef47	5	1	50	98
Ef44	5	2	50	96
Ef39	2	0	20	100
Ef36	5	0	50	100
Ef29	3	1	30	98
Ef26	4	2	40	96
Ef23	1	0	10	100
Ef16	4	2	40	96

* *The number in each protein band designation represents the apparent molecular mass in kilodaltons. IE, infective endocarditis*.

Immunoblotting performed with the 10 *S*. *gallolyticus* IE sera showed reactivity to 15 antigenic bands (Table 2). Each patient showed reactivity to at least two bands. When compared with immunoblot performed using 40 sera from patients without *S. gallolyticus* IE, we identified two bands, Sg97 and Sg65, with a specificity of 95% and three bands, Sg116, Sg75, and Sg59, with a sensitivity of 100%. Among blood donors, 13/17 sera showed reactivity to at least one antigenic band.

**Table 2:** WB pattern of reactivity of sera from *S. gallolyticus* infective endocarditis.

Protein band^*^	**Number of sera with WB reactivity to protein band**		**Sensitivity (%)**	**Specificity (%)**
	***S. gallolyticus* IE (*n* = 10)**	**Controls (*n* = 57)**		
Sg314	4	3	40	95
Sg232	2	2	20	96
Sg116	10	21	100	63
Sg97	7	7	70	88
Sg88	2	4	20	93
Sg75	10	14	100	75
Sg65	3	3	30	95
Sg59	10	15	100	74
Sg52	2	9	20	84
Sg42	3	4	30	93
Sg32	4	5	40	91
Sg29	5	4	50	93
Sg26	1	1	10	98
Sg25	2	6	20	89
Sg16	5	6	50	89

* *The number in each protein band designation represents the apparent molecular mass in kilodaltons. IE, infective endocarditis*.

**Figure 4:**
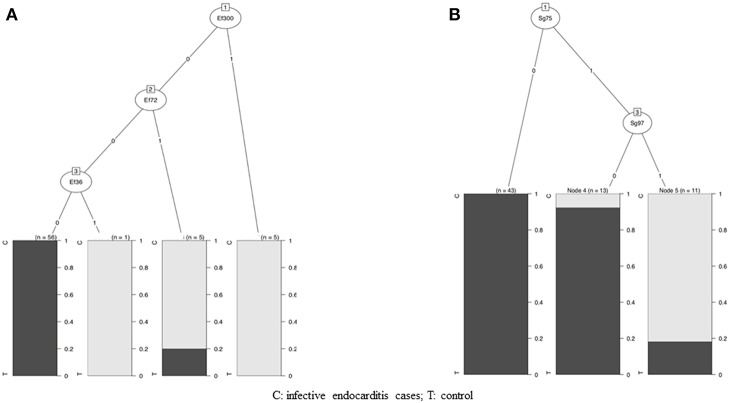
Algorithm to select discriminatory bands for the diagnosis of *E. faecalis* and *S. gallolyticus* IE. **(A)** Diagnosis algorithm for detection of *E. faecalis* IE by Western blotting (WB). The algorithm used reactivity to three protein bands: Ef300, Ef72, and Ef36. Depending on the presence or absence of reactivity to these protein bands, blots were classified as *E. faecalis* IE or not. **(B)** Diagnosis algorithm for detection of *S. gallolyticus* IE by WB. The algorithm used reactivity to two protein bands: Sg75 and Sg97. Depending on the presence or absence of reactivity to these protein bands, blots were classified as *S. gallolyticus* IE or not.

Based on these results, we created, using the CHAID method, an algorithm to select discriminatory bands for the diagnosis of *E. faecalis* and *S. gallolyticus* IE (Figure 4).

For *E. faecalis* IE, the algorithm used the reactivity to the three antigenic bands Ef300, Ef72, and Ef36. Concerning *S. gallolyticus* IE, the algorithm used reactivity to the two antigenic bands Sg97 and Sg75.

### Scoring Method for *E. faecalis* and *S. gallolyticus* IE Diagnosis Using WB

We propose the following criteria for the diagnosis of *E. faecalis* and *S. gallolyticus* IE: first, the presence of reactivity to at least two antigenic bands for *E. faecalis* or *S. gallolyticus* antigens. Then, according to the established algorithm, positivity to at least one of the proteic bands Ef300, Ef72, or Ef36 for *E. faecalis* and positivity for the two proteic bands Sg75 and Sg97 for *S. gallolyticus*.

### Evaluation of WB as a Diagnostic Method on a Prospective Cohort

From March 2018 to November 2018, we evaluated our scoring system prospectively and blindly on 363 sera obtained from patients with clinical suspicion of IE. All patients benefited from our standardized diagnostic procedure and *E. faecalis* and *S. gallolyticus* immunoblotting. The final diagnosis was made using our standardized procedure and ESC criteria. IE was excluded for 270 patients (including six patients with *E. faecalis* bacteremia without IE) and 93 had a diagnosis of definite IE, including 79 patients with positive blood cultures (11 *E. faecalis* IE, 4 *S*. *gallolyticus*, 27 *S*. *aureus*, 26 viridans group streptococci, and 11 other microorganisms) and 16 with BCNE (2 *B. henselae* IE and 14 without etiology).

Using our immunoblotting diagnosis criteria, 17 blots were in favor of a diagnosis of *E. faecalis* IE compared to 20 blots compatible with *S. gallolyticus* IE. This diagnostic scheme allowed us to identify all cases of proven *E. faecalis* and *S. gallolyticus* IE. All six *E. faecalis* bacteremia without IE had a negative immunoblot.

However, among the 17 immunoblots in favor of *E. faecalis*, four false positives corresponding to three IE (one *E. faecium* IE, one *B. henselae* IE, and *Haemophilus parainfluenzae* IE) and one patient with *E. faecalis* prostatitis (*E faecalis* isolated in urine, negative blood cultures) were identified.

Among the 20 immunoblots in favor of *S. gallolyticus* IE, 14 false positives corresponding to 5 IE (one *H. parainfluenzae* IE, one *S*. *mutans* IE, one *S. mitis* IE, one *S. oralis* IE, and one *E. faecalis* IE) and 9 patients with no IE diagnosis were identified.

In this prospective cohort, *E. faecalis* immunoblotting was used to diagnose *E. faecalis* IE with a sensitivity of 100% and a specificity of 99%. The positive predicted value was 73% and the negative predictive value was 100%.

Regarding *S. gallolyticus* infection, immunoblot had a sensitivity of 100% and a specificity of 95%. However, the positive predicted value was 22%, whereas the negative predictive value was 100%.

Based on immunoblotting results, we identified 4 potential etiologies in the 14 BCNE cases with no identified pathogen. A 48-year-old male (intravenous drug user) with tricuspid valve IE had a reactivity profile suggesting a diagnosis of *S. gallolyticus* IE. Positron emission tomography (PET/CT) was not performed. An 89-year-old man exhibited a reactivity profile suggesting *E. faecalis* IE. This patient had a BCNE on pacemaker leads and the PET/CT showed focal colonic hypermetabolism. A third patient showed a reactivity profile in favor of both *E. faecalis* and *S. gallolyticus* IE. A 70-year-old man had a native aortic valve IE. He suffered from cirrhosis with portal hypertension and esophageal varices. He had been treated with antibiotics for a pneumonia and had a colonoscopy with cecal polyp resection 2 weeks before IE diagnosis. The histology of the aortic valve after cardiac surgery confirmed the diagnosis of IE and showed Gram-positive cocci. Finally, a 74-year-old man with a bronchial adenocarcinoma had a marantic mitral valve endocarditis and positive immunoblot blot profile in favor of *S. gallolyticus* IE. The PET scanner showed a highly suspect hypermetabolic pulmonary mass and bone lesions compatible with metastasis.

### Analysis of Discordant Cases

We focused on the patients for whom *E. faecalis* WB yielded false positives. For the patient with *E. faecium* IE, WB with *E. faecalis* and *E faecium* antigens was performed and cross-reaction was observed with both antigens. For the patient with *B. henselae* IE, we controlled the WB; it was only positive for *B. henselae* and negative for *E. faecalis*. For the patient with *H. parainfluenzae* IE, we performed a WB with *E. faecalis, S. gallolyticus*, and *H parainfluenzae* (with the strain isolated from the patient's blood) and a cross-reaction was observed with both *S. gallolyticus* and *H. parainfluenzae*.

## Discussion

In BCNE, diagnosis of the causal pathogen remains essential due to its impact on antibiotic treatment and portals of entry, especially for *E. faecalis* and *S. gallolyticus* and their possible association with colorectal cancer (Corredoira et al., 2015). Over the past few years, we have diversified the diagnostic tests used for the diagnosis of BCNE (Houpikian and Raoult, 2003; Raoult et al., 2005; Fournier et al., 2011, 2017) but new tools are still needed. In this study, we evaluated WB as a tool for the diagnosis of BCNE without etiology. We used the four antigens *E. faecalis, S. gallolyticus, S. anginosus*, and *S. aureus*, which represent some of the most frequent etiological agents of IE and BCNE diagnosed only by valve PCR. Our technique was effective to detect the antigenic profiles of the four tested agents, but cross-reactions were frequent for *S. aureus* and *S. anginosus*. For *E. faecalis* and S*. gallolyticus* IE, we identified reactivity to 14 and 15 antigenic bands, respectively. All patients with IE caused by one of these two pathogens exhibited reactivity to at least two proteic bands. We created a diagnostic algorithm for *E. faecalis* and *S. gallolyticus* IE using reactivity to antigenic bands showing the best specificity (the three proteic bands Ef300, Ef72, and EF36 for *E. faecalis*, and the two proteic bands Sg97 and Sg75 for *S. gallolyticus*). When we evaluated the diagnostic performance of our scoring method, we observed a good performance for *E. faecalis* IE with a PPV of 73% and an NPV of 100%, but a lower performance for *S. gallolyticus* IE (NPV 100%, PPV 22%). In addition, the evaluation of this technique in a prospective cohort showed the ability to identify all cases of *E. faecalis* IE. We found only two false positives in a patient with *E. faecalis* prostatitis and another one with *E. faecium* IE. Moreover, among the patients without IE, the five patients with bacteremia had negative immunoblots. Other studies demonstrated that immunoblotting was able to distinguish patients with *E. faecalis* deep-seated infections from patients with isolated bacteremia (Sulaiman et al., 1996).

Our results are concordant with the literature. In the 1980s, studies indicated that WB or enzyme-linked immunosorbent assay (ELISA) could be useful for the diagnosis of enterococcal endocarditis, but this seems to have been forgotten (Burnie et al., 1987; Burnie and Clark, 1989). Other studies have described cross-reactions between *E. faecalis* and *E. faecium* (Burnie et al., 1987) or between *S. gallolyticus* and *E. faecalis* using indirect ELISA (Burnie and Clark, 1989). However, cross-reactions between *Bartonella* sp. and *E. faecalis* were unknown and unexpected as these bacteria belong to distinct phyla. As a consequence, for patients with BCNE and a positive *Bartonella* sp. WB, a WB for *E. faecalis* should systematically be performed when no other proof of *Bartonella* infection is obtained.

Regarding *S. gallolyticus*, the performance of our diagnostic criteria is poorer. However, we showed that this technique made it possible to identify all IE cases caused by this species. This is interesting since *S. gallolyticus* is a common agent of BCNE in developed countries (Fournier et al., 2017). Also, several false positives were observed, particularly in patients with IE caused by other *Streptococcus* sp. WB was used to evaluate the immune response to *S. gallolyticus* in patients with adenomatous polyps in the colon (Garza-González et al., 2012) and detected two prominent immunogenic proteins that may be promising serological markers for the presence of adenomatous polyps.

This study suggests that *E. faecalis* and *S. gallolyticus* WB performed systematically during the diagnosis process of BCNE could lower the incidence of cases without etiological pathogen identified. This could lead to important clinical consequences such as indication of colonoscopy to look for polyps or colon cancer, in case of positivity for one of these two pathogens. In order to obtain a most reproducible diagnosis, we performed a simple diagnostic algorithm for each pathogen. These results should be confirmed by further studies but this method could be expanded to other centers who have experience in BCNE diagnosis. This technique could be easily developed in a microbiological laboratory that has the opportunity to design WB technique.

We acknowledge the fact that our study has certain limitations. This is a preliminary study with a small number of IE cases, particularly few *S. gallolyticus* and BCNE IE. This study was also carried out in a reference center for the management of IE, which does not reflect the diagnostic methods performed in other centers. The diagnostic approach described here needs to be generalized to a larger prospective cohort in order to confirm the results.

In conclusion, using WB constitutes a promising method to obtain a specific diagnosis of *E. faecalis* or *S. gallolyticus* IE in the absence of positive blood cultures.

## Data Availability

The datasets generated for this study are available on request to the corresponding author.

## Ethics Statement

The study and the cases reports form were approved by the local and national institutional review boards and ethics committees (2012-A01549-34).

## Author Contributions

FA performed the Western blot, collected the data, and wrote the *Materials and Methods* section and *Discussion* of the manuscript. FG supervised FA, managed the patients, collected the data, and wrote the introduction and discussion of the manuscript. BA performed the Western blot. SE managed the serological diagnosis of the two cases. HC performed the statistical analysis. J-PC managed the patients and collected the data. GH managed the patients and collected the data. P-EF corrected the manuscript. DR designed the manuscript.

### Conflict of Interest Statement

The authors declare that the research was conducted in the absence of any commercial or financial relationships that could be construed as a potential conflict of interest.
